# The impact of maturity on the ability of *Eimeria acervulina* and *Eimeria meleagrimitis* oocysts to sporulate

**DOI:** 10.1051/parasite/2021031

**Published:** 2021-04-02

**Authors:** Jean-Michel Répérant, Martine Thomas-Hénaff, Chantal Benoit, Pierre Le Bihannic, Nicolas Eterradossi

**Affiliations:** 1 Avian and Rabbit Virology, Immunology, Parasitology Unit, ANSES, Laboratory of Ploufragan-Plouzané-Niort PO Box 53 22440 Ploufragan France

**Keywords:** *Eimeria*, Coccidia, Sporulation, Oocysts, Chicken, Turkey

## Abstract

The sporulation of oocysts of *Eimeria* that infect poultry is known to be under the influence of environmental conditions, including temperature, oxygen supply, and moisture. However, even when these conditions are optimal, the level of sporulation can remain low. The effect of oocyst maturity on their ability to sporulate was investigated for two species of *Eimeria*: *E. acervulina* of chickens, and *E. meleagrimitis* of turkeys. After oral infection of birds, oocysts were collected at their production site in the intestine at different times around the prepatent period. The percentage of sporulation was determined by observation of 100 oocysts for each sample. With *E. acervulina*, it was observed that sporulation depended on the time of collection of the oocysts in the intestine, and that it increased with aging oocysts (from 5% to 40% globally in 8 h). With *E. meleagrimitis*, sporulation remained low with oocysts collected in the duodenum (below 20%), but oocysts collected in the midgut and in the lower intestine sporulated more efficiently (around 80%) than oocysts collected in the duodenum at the same time. One explanation for these results is the assumption that oocysts may be produced before fertilization, and that microgametes have not yet fertilized the newly produced oocysts. As time goes on, more oocysts would be fertilized, locally in the duodenum for *E. acervulina*, and descending along the gut for *E. meleagrimitis*. This hypothesis needs to be investigated further, but it could lead to new approaches to control these parasites by targeting the microgametes.

## Abstract (fr)

La sporulation des oocystes chez les *Eimeria* qui infectent la volaille est connue pour être influencée par les conditions environnementales (température, apport d’oxygène et humidité). Cependant, même lorsque ces conditions sont optimales, le niveau de sporulation peut rester faible. L’effet de la maturité des oocystes sur leur capacité à sporuler a été étudié pour deux espèces d’*Eimeria* : *E. acervulina* du poulet et *E. meleagrimitis* de la dinde. Après infection orale des oiseaux, les oocystes ont été collectés sur leur site de production dans l’intestin à différents moments autour de la période prépatente. Le pourcentage de sporulation a été déterminé en observant 100 oocystes pour chaque échantillon. Avec *E. acervulina*, il a été observé que le pourcentage de sporulation dépendait du moment de la collecte des oocystes dans l’intestin, et qu’il augmentait avec des oocystes vieillissants (globalement de 5 % à 40 % en 8 heures). Avec *E. meleagrimitis*, le pourcentage de sporulation restait faible avec les oocystes collectés dans le duodénum (inférieur à 20 %), mais les oocystes collectés dans l’intestin moyen et dans l’intestin inférieur ont sporulé plus efficacement (environ 80 %) que les oocystes recueillis dans le duodénum en même temps. Une explication de ces résultats repose sur l’hypothèse que les oocystes peuvent être produits avant la fécondation et que les microgamètes n’ont pas encore fécondé les oocystes nouvellement produits. Avec le temps, davantage d’oocystes seraient fécondés, localement dans le duodénum pour *E. acervulina*, et descendant le long de l’intestin pour *E. meleagrimitis*. Cette hypothèse doit être approfondie, mais elle peut conduire à de nouvelles approches pour contrôler ces parasites en ciblant les microgamètes.

## Introduction

Coccidia belonging to the *Eimeria* genus are obligate parasites of a wide variety of hosts. Their life cycle is well known for most stages (including excystation), and schizogony and conditions for sporulation in the environment have been characterized. Gamogony, the reproductive sexual stage of the life cycle, which is characterized by the formation of female macrogamonts and male microgamonts, leads to the formation of oocysts after fertilization processes [5, 16].

During gamogony, little is known about fertilization. It is believed to occur between microgametes and macrogametes, although no observations have confirmed this. After fertilization, the oocysts are released and pass into the environment through droppings. At that time, they contain one single cell, the sporont, and are not yet infective. They need to evolve to become infective. This maturation stage, called sporogony, involves division of the sporont, resulting in an oocyst containing four sporocysts, each containing two infective sporozoites. Specific environmental conditions are needed for oocysts to sporulate, such as oxygen availability, optimal temperature, and moisture [2]. However, even when optimal conditions are present, oocysts may sporulate poorly, or not sporulate at all, as has been observed after treatment of infected birds with certain anticoccidial drugs [10, 11, 13, 14].

The aim of this study was to investigate the sporulation ability of oocysts for two *Eimeria* species of chickens and turkeys, depending first on the degree of maturity (time from the moment of appearance) of the oocysts, and second on their site of development in the gut. *Eimeria acervulina*, which is the most prevalent species in chickens [7, 17], and *Eimeria meleagrimitis*, which is not only the most frequent species in turkeys but also has a major impact on growth and health on turkey farms, were chosen for this study. A better understanding of the factors that influence the sporulation process could unveil new strategies to reduce or prevent sporulation in order to control these parasites and their impact on their avian hosts.

## Materials and methods

### Ethics statement

All experiments were performed in agreement with national regulations on animal experiments and animal welfare, according to authorizations Nu22-4 and B-22-745-1 by the *Préfecture des Côtes d’Armor*, France. Experiments were subject to prior approval by the authors’ Institutional Ethics Committee (Approved protocol 4710-2016032517572396 v2 by Cometh Anses/National Veterinary School of Alfort, France/University of Paris Est – Créteil, Val de Marne, France, registered in France as number 16 by Comité National de Réflexion Éthique sur l’Expérimentation Animale).

### Chickens

Conventional cockerels of the ISA-Brown strain were obtained from a commercial hatchery (ISA S.A.S., Hendrix Group, Mur de Bretagne, France) at hatching day and were grown free of coccidia in wired floor cages, until the experiments were performed under controlled conditions in containment facilities (Anses, Ploufragan, France).

### Turkeys

Conventional turkey poults of the Hybrid Grade Maker strain were obtained from a commercial hatchery in Brittany (Grelier France Accouvage, Hendrix Group, Plouguenast, France). They were grown in facilities free of coccidia (Anses, Ploufragan, France) in wired floor cages under controlled conditions.

### Coccidia

Two species of coccidia developing in the upper part of the small intestine were chosen, one specific to chickens: *Eimeria acervulina*, and one specific to turkeys: *Eimeria meleagrimitis*.

One pure strain (PA3 strain) and one field isolate (no. 7) of *E. acervulina* and one pure strain of *E. meleagrimitis* (PM3 strain) were used:

- Pure strains were originally purified from field samples by isolating one single oocyst and multiplying it in coccidia-free birds. Since their initial isolation (in 1999 for *E. meleagrimitis* and in 2000 for *E. acervulina*), the pure strains have been passaged twice a year in coccidia-free birds to maintain their viability.
- The field isolate of *E. acervulina* was obtained from droppings of a flock of chickens positive for coccidia. Oocysts were purified by flotation in saturated salt solution. Briefly, droppings were mixed with tap water and passed through a 250 μm filter. The suspension was centrifuged (3000 rpm for 7 min) and the pellet was resuspended in a salt saturated solution (excess of NaCl in water – density 1.18). The suspension was centrifuged at 1800 rpm for 7 min, and the supernatant was collected and washed three times in water with centrifugations at 3000 rpm for 7 min. After the last centrifugation, the pellet was suspended in a 2% potassium dichromate solution, observed under the microscope for the presence and cleanliness of oocysts. Then the oocysts were incubated for three days at 28 °C to sporulate. The oocyst suspension was administered orally to chickens (about 10,000 sporulated per bird) to obtain fresh oocysts. After the purification and sporulation process at 28 °C for three days in 2% potassium dichromate on agar, the oocysts were inoculated to new birds and the duodenal contents (which should contain mainly oocysts of *Eimeria acervulina*, as this species develops in the duodenum and jejunum, whereas other species – except *Eimeria praecox* are found below the duodenum) were collected 103 h after inoculation. The collected oocysts were observed under a microscope for size and shape homogeneity and to verify the absence of *Eimeria praecox*, which has oocysts that are larger than those of *Eimeria acervulina*, and the aspect of the gut mucosa was observed for typical lesions. Oocysts were incubated at 28 °C for three days on a semi-solid medium with potassium dichromate, and then stored at 4 °C until use.

### Inoculations

At 22 days of age, the birds were transferred to an experimental containment room, one day before inoculation, for acclimatization. They were inoculated on the next day, by oral gavage with an esophagus cannula, with 10,000 sporulated oocysts (1 mL).

### First study with collection of oocysts and measurement of sporulation levels

For the collection of oocysts of *E. meleagrimitis* in turkeys, each hour from 118 hours post-infection (pi) to 126 h pi, one bird was humanely euthanized (by electronarcosis immediately followed by decerebration and exsanguination) and the oocysts were collected at the duodenal level, by scraping the mucosa. Microscopic observation was performed to verify the presence of oocysts. The scraped samples were homogenized in a 2% potassium dichromate solution and the suspensions were incubated at 28 °C. After 3 days and 11 days, the sporulation rate was measured by directly counting under the microscope 100 oocysts and noting their sporulation status, except when the suspensions contained very few oocysts, in which case all observed oocysts were taken into account.

A second study was performed with *E. meleagrimitis* on turkeys kept under the same conditions as previously described. In this second study with turkeys, scrapings were collected at three anatomical locations in the intestine: duodenum, midgut (around Meckel’s diverticulum), and lower ileum. A total of 3 birds were used at each of 4 time points around the prepatent period (118, 121, 124 and 127 h pi). The oocysts were incubated at 28 °C and the sporulation rate was observed after three days.

For the collection of oocysts of *E. acervulina*, two studies were conducted. In the first one, using *E. acervulina* PA3 strain, oocysts were harvested each hour between 87 and 96 h pi, as described for *E. meleagrimitis*. In the second one, using *E. acervulina* isolate no. 7, oocysts were collected hourly from 112 to 118 h pi, and at a final time point at 119.5 h.

A third study was performed with *E. acervulina* PA3 strain with 30 chickens submitted to a similar experimental design. At 88, 90, 92, 94 and 96 h pi, five birds were used and oocysts were collected and incubated separately for each bird. Sporulation was measured after 3 and 10 days after collection of the oocysts and incubation at 28 °C, as previously described. Results of percentages of sporulation at the five time points of collection were statistically analysed using R-Software Kruskal–Wallis and Mann–Whitney tests. The Kruskal–Wallis test is a global non-parametric test that gives information on global significant differences, and the Mann–Whitney test allows pairwise comparisons.

In the studies performed with *E. meleagrimitis* and the first study with *E. acervulina* PA3 strain, additional birds were kept one more day to collect droppings with oocysts. These oocysts were placed in the same conditions to sporulate. In the second study conducted with *E. acervulina* (isolate no.7) and in the third one (with *E. acervulina* PA3 strain), there were no additional birds to collect the droppings and investigate the sporulation rate in the droppings.

## Results

### *Eimeria meleagrimitis* in turkeys, in the duodenum

**Figure 1:**
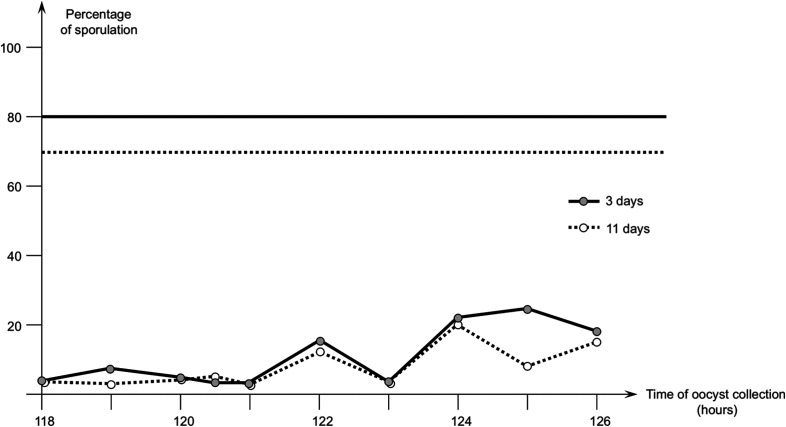
First study with *Eimeria meleagrimitis* – evolution of the percentage of sporulation of oocysts of *E. meleagrimitis* (PM3 strain) collected in the duodenum from 118 to 126 hours after infection. Percentage of sporulation was evaluated after incubation for 3 or 11 days at 28 °C. The horizontal straight lines show the percentage of sporulation observed among oocysts collected in droppings 144 h after infection.

The first study was conducted with sampling every hour between 118 and 126 h pi. The results of sporulation are shown in Figure 1.

Sporulation level was low (between 3 and 7% from 118 to 121 h pi), and slightly increased at 122 h (15%), and 124 h and up (18–25%). In contrast, sporulation of oocysts obtained from droppings collected at 144 h pi was much higher (80% sporulation after 3 days and 70% after 11 days). No difference in sporulation was obtained after incubation of oocysts for 3 days or 11 days, except for oocysts collected at 125 h, which had a percentage of sporulation lower after 11 days than after 3 days.

### *Eimeria meleagrimitis* in turkeys, oocysts sampled at different locations in the gut

**Figure 2:**
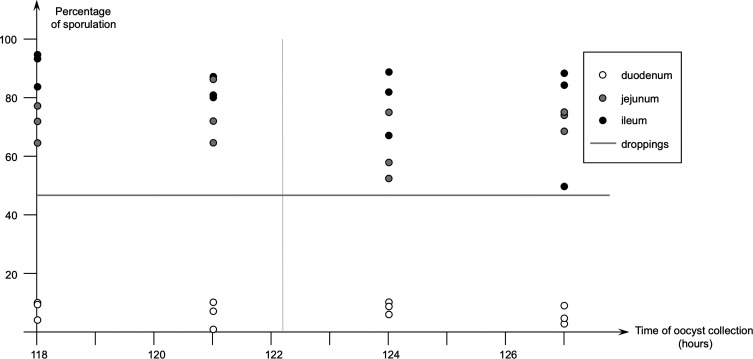
Second study with *Eimeria meleagrimitis* - percentage of sporulation depending on time of oocysts of *E. meleagrimitis* (PM3 strain) collected in three birds at each time in the duodenum, midgut and lower ileum (white, grey and black dots, respectively), compared with percentage of sporulation of oocysts collected in the droppings 144 h after infection (horizontal line)

Results of the second study performed with turkeys and collection of oocysts at different locations in the gut are shown in Figure 2. Like the first study performed with *E. meleagrimitis*, the level of sporulation was low (around 10%) with oocysts collected in the duodenum at 118, 121, 124 and 127 h pi. Oocysts collected in the midgut and in the lower ileum had a sporulation percentage above 50%. The percentages of sporulation did not appear to evolve in time in any part of the gut sampled. The percentage of sporulation in the droppings collected 144 h pi was around 50%.

### *Eimeria acervulina* in chickens, oocysts sampled in the duodenum

**Figure 3:**
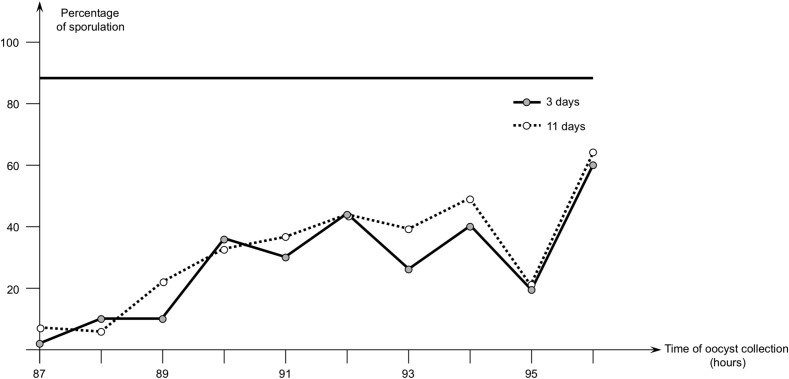
First study with *Eimeria acervulina* – evolution of the percentage of sporulation of oocysts of *E. acervulina* (PA3 strain) collected in the duodenum from 87 to 96 h after infection. Percentage of sporulation was evaluated after 3 and 11 days of incubation at 28 °C. The horizontal straight line shows percentage of sporulation as observed in oocysts collected from droppings after 3 days of incubation at 28 °C.

In the first study, the birds were euthanized from 87 to 96 h pi. Oocysts were obtained at each time point, and the results of sporulation are presented in Figure 3. From 87 to 89 h, the sporulation rate was between 2 and 10%, then it increased in oocysts collected from 90 to 95 h, to reach 19–44% sporulation. At 96 h pi, sporulation percentage was 60% for oocysts collected in the duodenum, whereas it reached 90% for those collected from droppings.

**Figure 4:**
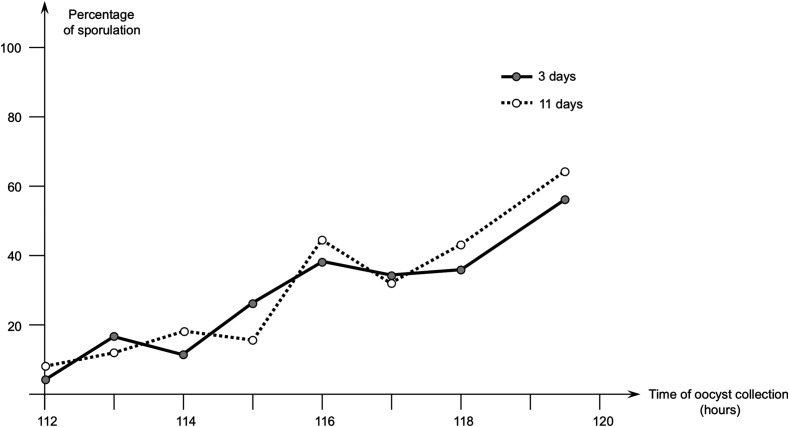
Second study with *Eimeria acervulina* - evolution of the percentage of sporulation of oocysts of *E. acervulina* (isolate no. 7) collected in the duodenum from 112 to 119.5 h after infection. Percentage of sporulation was evaluated after 3 and 11 days of incubation at 28 °C.

The second study was performed with the field isolate named no. 7. For this isolate, oocysts were produced later than with the PA3 strain, and oocyst sporulation from 112 to 119.5 h pi was observed. The sporulation rate for oocysts collected in the duodenum progressed regularly starting from 4% at 112 h pi to reach 56% at 119.5 h pi (Fig. 4).

The results of the third study with the laboratory strain of *E. acervulina* are shown in Table 1. At 88 h pi, percentage of sporulation ranged between 2.6 (after 3 days) and 5.4% (after 10 days). At 90 and 92 h pi, the percentage of sporulation was under 10%, then it increased at 94 and 96 h pi to reach 20–36% of sporulated oocysts. The sporulation in droppings was 34%. Differences in percentage of sporulation were significant between 88 h pi and 94 and 96 h pi, after 3 days of sporulation or 10 days.

**Table 1:** Third study with *E. acervulina* – evolution of the percentage of sporulation of oocysts of *E. acervulina* (PA3 strain) collected in the duodenum every two hours from 88 to 96 h after infection. One hundred oocysts were observed for each sample. Each value is the mean value of five birds (corresponding individual values are between parentheses). Percentage of sporulation was evaluated after incubation for 3 or 10 days at 28 °C. The percentage of sporulation of oocysts collected in droppings is given in the last line of the table. Different letters in superscript in a same column mean statistically significant differences (Kruskal–Wallis test followed by Mann–Whitney test).

Hours post infection	Percentage of sporulation of oocysts	
	3 days	10 days
88	2.6^a^	5.4^a^
	(1–2–2–3–5)	(3–4–4–6–10)
90	4^a,b^	9^a,b^
	(2–2–4–6–6)	(0–2–5–11–27)
92	8^b,c^	8^a^
	(2–3–13–14–18)	(3–4–4–12–17)
94	11^c^	19^b,c^
	(7–9–12–12–15)	(14–16–19–21–25)
96	20^c^	36^c^
	(13–17–20–20–25)	(23–29–36–39–53)
In droppings		34

## Discussion

The prepatent period of *E. meleagrimitis* differs depending on the authors. For instance, according to Reid [12] it is 103 h, and Long *et al*. [9] also found a prepatent period of 103 h. However, according to Vrba & Pakandl [15] and El-Sherry *et al*. [3], the prepatent period lasts 120 h. As we did not know the prepatent period of the PM3 strain, a preliminary study on turkeys was performed with samples collected in the duodenum between 98 and 101 h pi, in order to collect the first oocysts produced in case of a prepatent period of 103 h. No oocyst was observed in the mucosal scrapings at these times. The time points of sampling for the first study with *E. meleagrimitis* were defined between 118 and 126 h pi, to encompass the prepatent period of 120 h.

Concerning oocyst production of *E. acervulina*, a delay was observed for the isolate no. 7 compared to the PA3 strain, the prepatent period of which was compatible with 97 h described in the literature. However, typical lesions of *E. acervulina* were observed with isolate no. 7, indicating it was definitely *E. acervulina*. This isolate may have been slower to complete its life cycle, or the storage period may have influenced the prepatent period, as shown for *Eimeria tenella* [1].

Little difference was observed between the results of sporulation after incubation at 28 °C for 3 and 11 days (or 10 days in the last experiment). These minor differences may be due to variation in sampling and counting:

1. These results show that the sporulation ability of *E. acervulina* increases with time for young oocysts collected at their site of production in the gut. Although oocyst sporulation did not increase regularly, and even sometimes remained the same for several consecutive hours, the sporulation ability of oocysts harvested in the duodenum increased with time in all studies.
2. For *E. meleagrimitis*, the results are different and suggest that sporulation does not increase with time in the duodenum, but sporulation ability increases during travel of the oocysts along the intestine.
3. This work strongly suggests that factors other than those already known (such as environmental parameters: temperature, moisture, and oxygen supply) play a role in the intestine and affect sporulation. For *E. acervulina*, it seems that these factors act at the duodenum location, and for *E. meleagrimitis*, they would play a role lower in the gut. However, which factors and mechanisms are involved are not known.
4. Perhaps factors targeting the oocyst physically are involved such as pH, chemical interactions, or interactions with bacteria. However, we did not observe damaged oocyst, and oocysts that did not sporulate after incubation for 3 or 11 days had a normal aspect. Another explanation might be the absence or inhibition of fertilization. Some studies have shown that oocysts of *Toxoplasma*, another coccidian parasite, can be produced without fertilization, but at a minor level [4]. In a similar way, a correlation was observed between reduced microgamete numbers (following prolonged culturing at sub-standard temperatures) and poor sporulation rates in oocysts [8]. Furthermore, oocyst wall formation is initiated while the macrogamete is still within the host cell [5, 6, 16]. Thereafter, if fertilization occurs between the macrogamete and microgamete, microgametes must get inside the host cell to reach the macrogamete. Another possibility could be that fertilization takes place only when the oocyst wall is formed and the female gamete is outside the host cell, with microgametes entering the oocyst via the micropyle. Ferguson [5] noticed that microgametes are mainly found in contact with oocysts and rarely next to macrogametes, suggesting that fertilization could occur between microgametes and oocysts. In our experiments, microgametes were observed alongside with oocysts at each time of sampling, strengthening this hypothesis of fertilization between microgametes and oocysts.

Moreover, the effect of some anticoccidial drugs that cause low sporulation rates could be explained by a direct action on the microgamete which could not fertilize the oocyst.

In our observations, early produced oocysts may not be fertilized yet, and hence cannot sporulate. As time passes, more oocysts could be fertilized by microgametes and the percentage rate would thus increase.

Concerning *E. meleagrimitis*, microgametes could meet the oocysts later, during travel of the oocysts at the end of the duodenum or in the jejunum, as suggested by the fact that oocysts going down the intestine are more able to sporulate. Of note, in low infections, *E. acervulina* is only located in the duodenum, but *E. meleagrimitis* develops in the whole small intestine, but gamonts are more numerous in the jejunum and ileum [3]. Thus, oocysts of *E. meleagrimitis* produced in the duodenum could be less exposed to microgametes, and the ability to sporulate would not evolve in time at that location.

5 – The demonstration of such a mechanism or of the influence of intestinal factors on sporulation would be an important improvement in our understanding of the fertilization process and suggests it might be interesting to develop control tools targeting the microgamete. Indeed, the microgamete has not been well studied since the description of the life cycle of *Eimeria*, and it is not the target of current methods used to control coccidia development. We are working on this hypothesis in order to further study the capacity of microgametes to fertilize young oocysts.
